# Supplementary material for: Collaborative intelligence in AI: Evaluating the performance of a council of AIs on the USMLE
Source: PLOS Digit Health. 2025 Oct 9;4(10):e0000787. doi: 10.1371/journal.pdig.0000787 (PMC12510544; doi:10.1371/journal.pdig.0000787)
Supplement: S2 File — (ZIP) [file pdig.0000787.s002.zip › S2 Data Raw data files containing unprocessed question inputs and LLM outputs including transcripts of deliberations/STEP3_Question-9-1_Type-MC-NJ.docx]

**Facilitator** **[Round 1]:** A 35-year-old man, who has been your patient for the past year, comes to the office to request that a copy of his medical records be sent to another physician in town. The patient has an overdue account balance of $750, which he has not paid for several months. He signs an office form authorizing release of health information and provides the name and address of the other physician to whom he would like the records sent. It is most appropriate to inform this patient of which of the following regarding his request for release of his medical records? (A) They will be released to his new physician promptly (B) They will be released when he makes a payment on his account (C) They will be released when he pays his balance in full (D) They will be released when his new physician contacts your office and requests them

**AI_A:** RESTATING THE QUESTION: To paraphrase, what's the best course of action when a patient with an overdue account balance requests the transfer of his medical records to another physician? We need to consider options A) Quick transfer, B) Transfer after any payment, C) Transfer only when the full balance is cleared, or D) Transfer once the new physician makes a request. PLANNING: Assumptions here include the idea that the release of medical records should be conditional on payment, or that the delay in transfer would motivate the patient to settle his account. An alternate assumption, one rooted in medical ethics, is that a patient's financial obligations should not hinder access to their medical records - these records are essential for continuity of care. CLINICAL REASONING: Upon evaluating, it's ethical to prioritize the patient's health care needs over financial issues. The new physician will need the patient's medical history for effective management. Holding the records due to unsettled bills could interrupt the continuum of care, therefore jeopardizing the patient's health. Furthermore, it's important to note that, under HIPAA laws in the U.S., health care providers are obliged to provide patients with their own health information upon request, regardless of account standings. CONCLUDING: Given these considerations, it's ethically and legally appropriate to send these records promptly to the patient's new physician. Even though the patient may be indebted, the protection and quality of health care should always take precedence over financial concerns. RESPONSE: The most appropriate course of action is to inform the patient that his documents will be dispatched to his new doctor in a timely manner [(A) They will be released to his new physician promptly], ensuring the quality and continuity of his medical care.

**AI_C:** RESTATING THE QUESTION: This scenario is about a man, aged 35, who is a patient of yours. He wants to transfer his medical records to another doctor. However, he is behind on his account and owes $750. He has filled out a form to provide approval for sharing his health details and named the other physician. The issue here is to decide under what circumstances his medical documents should be released. PLANNING: This situation revolves around ethics, patient rights, and laws related to health information privacy, specifically the Health Insurance Portability and Accountability Act (HIPAA). The assumption may be to withhold the patient's records until the debt is settled. Altering this assumption, we consider the legality and ethicality of the patient's rights to his medical information irrespective of his pending bill. CLINICAL REASONING: Legally and ethically, patients have a right to their healthcare records. HIPAA stipulates that healthcare providers cannot deny patients access to their own health records because of unpaid bills. This is based on the understanding that healthcare is a fundamental right and that information about one's own health should not be contingent upon financial status. Accessibility to health records enables patients to take an active part in managing their health, promoting patient-centered care. CONCLUDING: Therefore, the consideration is not about the patient's debt but rather, it's about the healthcare provider's obligation to promptly forward the patient's medical records as requested. A patient's right to access his own health records, as protected by federal law, overrules financial disputes. RESPONSE: Answer (A) They will be released to his new physician promptly. This respects his rights as a patient and complies with the Health Insurance Portability and Accountability Act.

**AI_E:** RESTATING THE QUESTION: The situation presented here involves a male patient who has an overdue medical bill of $750. Despite holding an outstanding balance, the patient wishes to transfer a copy of his medical records to another physician. The question here is, what action should take place regarding the release of the patient's medical records considering his current financial status? PLANNING: The assumption made in this scenario is that payment or eradication of debt is a requirement for the release of the patient's health information. However, there might be a separate assumption in which the patient's grievances are addressed, and this topic can be reframed around the ethics and legalities of health information disclosure. The legal and ethical instruments would guide us in terms of transparency, accountability, good governance, and efficiency should be accompanied by justice, beneficence, preserving autonomy and therapeutic fidelity. Therefore, understanding medical law will clear this assumption which should be taken as top priority. CLINICAL REASONING: From a clinical perspective, it's important to understand that any discussions around remittances or repayments shouldn't eclipse the overarching purpose of health care, which is securing the wellbeing of the patient. Health information, like medical records, significantly influence the continuity, coordination, and quality of care. Thus, transferring these records promptly would ensure that the new physician is well-informed about the patient's medical history, hence allowing for effective healthcare delivery. CONCLUDING: Conclusively, the potential drawbacks associated with delaying the release of the patient's medical records due to unpaid dues, such as the possible stall in healthcare continuity, significantly outweigh the benefits of immediate debt repayment. RESPONSE: Therefore, the most appropriate action to take would be to inform this patient that his medical records will be released to his new physician promptly (Option A). This course of action would uphold his rights as a patient while ensuring his wellbeing and continuity of care are not compromised.

**AI_B:** RESTATING THE QUESTION: What's under discussion fundamentally is an ethical and legal principle in medicine regarding the access and transfer of a patient's medical records. Despite the patient owing an amount of $750, he wants his records transferred to a new physician. The question is how to best handle his request considering the financial debt in the background. PLANNING: The assumptions here could be that (a) medical records can be withheld until debts are cleared, (b) records should be transferred promptly despite monetary issues, or (c) the request from the new physician should be awaited before releasing records. Of these, the first assumption assumes that health care access and information is conditional on financial status, which would deprive persons in financial difficulty from having continuity of care with a different physician. It would be ethically problematic to withhold information that could potentially impact medical management. The second assumption ensures unrestricted access to information which could be crucial to healthcare decisions. Lastly, the third assumption implies that patient's request isn't enough and a professional-to-professional communication is required to release the information, which could potentially delay care. CLINICAL REASONING: When prioritizing these assumptions, we need to take into account both ethical and legal considerations. Legally, the Health Insurance Portability and Accountability Act (HIPAA) guarantees patients' rights to their health information, irrespective of financial status or debt. Moreover, ethically, holding medical records hostage for payment could negatively affect patient care, and may not serve the best interest of the patient. CONCLUDING: Thus, from an ethical perspective prioritizing patient care, and from a standpoint of legal requirement by HIPAA, the assumption that patient's medical records should be promptly transferred to a new physician, regardless of any outstanding debt, is the best scenario here. RESPONSE: The appropriate action to inform this patient is that his medical records will be released to his new physician promptly (A). Any accounts or payments due should not interfere with continuity of medical care, and as such, the patient's records should be promptly sent to new physician, ensuring seamless care. Financial matters should be dealt separately from issues impacting patient care.

**AI_D:** (1) RESTATING THE QUESTION: The patient in question, a 35-year-old male, is asking for a copy of his medical records to be sent to a different medical practitioner. He has an outstanding account balance of $750 which he hasn't paid off for multiple months. Despite this debt, he has signed a form authorizing the release of his healthcare information and has provided the relevant contact details for the physician who should receive these records. We are being asked to select the most fitting action from a choice of possibilities regarding the release of his medical records. (2) PLANNING: The options include releasing the records promptly, releasing them after receiving some payment or full balance, or when the new physician requests them. All these assumptions prioritise differing ethical, legal, and practical considerations. The ethical considerations involve maintaining patient trust, respect for autonomy, and ensuring continuity of care; legal considerations involve jurisdiction-specific laws governing patient record sharing; practical considerations involve clinic-specific policies on debt repayment. (3) CLINICAL REASONING: Releasing the records promptly (A) ensures continuity of patient care and preserves patient autonomy and trust. However, it does not tackle the issue of the unpaid balance. Waiting for some payment (B) or the full balance (C) before releasing the records could be seen as a practical response to the issue of outstanding payment, but these options potentially threaten continuity of care and risk damage to the patient-provider relationship. Waiting for the new physician to request the records (D) might delay continuity of care, especially if the reason for the request is time-sensitive. (4) CONCLUDING: While all the options have their considerations, from a clinical reasoning perspective, it is crucial to ensure continuation of patient care and respect for autonomy. Laws surrounding healthcare often prioritize patient rights, including access to their medical records. (5) RESPONSE: Therefore, in line with these considerations, the most appropriate action would likely be to inform the man that his medical records will be released to his new physician promptly (A), regardless of his unpaid balance. It’s important to note that the issue of unpaid bills should be pursued separately and not obstruct the process of medical care.

**Facilitator** The consensus among the AI responses is that the most appropriate course of action is to promptly release the patient's medical records to his new physician (Option A). This decision is based on ethical, legal, and clinical considerations. Ethically, it is important to prioritize the patient's healthcare needs over financial issues, as withholding medical records due to unpaid bills could disrupt the continuity of care and potentially jeopardize the patient's health. Legally, under the Health Insurance Portability and Accountability Act (HIPAA) in the U.S., healthcare providers are obliged to provide patients with their own health information upon request, regardless of their financial status or outstanding debts. Clinically, the new physician requires the patient's medical history for effective management of his health. Therefore, despite the patient's overdue account balance, his right to access his health records and the continuity of his care should not be compromised. The issue of the unpaid balance should be addressed separately and should not obstruct the process of medical care.
